# Epitope-guided optimization improves the protective efficacy of NA-based mRNA vaccines against seasonal influenza A(H3N2) virus

**DOI:** 10.71150/jm.2609004

**Published:** 2026-10-31

**Authors:** Hyunbeen Kim, Sangyi Lee, Seunghye Cho, Woojin Shin, Soyoung Lee, Atanas V. Demirev, Taeyoung Lee, You-Jin Kim, Dokeun Kim, Sae Am Song, Kyung Ran Jun, Jin Il Kim

**Affiliations:** 1 Department of Microbiology, Institute for Viral Diseases, Korea University College of Medicine, Seoul 02841, Republic of Korea; 2 Division of Infectious Diseases Vaccine Research, Center for Vaccine Research, National Institute of Infectious Diseases, Korea National Institute of Health, Cheongju 28159, Republic of Korea; 3 Department of Lab Medicine, Haeundae Paik Hospital, Inje University College of Medicine, Busan 48108, Republic of Korea; 4 Vaccine Innovation Center, Korea University College of Medicine, Seoul 02841, Republic of Korea; 5 Biosafety Center, Korea University College of Medicine, Seoul 02841, Republic of Korea

**Keywords:** antigen design, epitope prediction, H3N2 influenza, mRNA vaccine, neuraminidase

## Abstract

Neuraminidase (NA) is increasingly recognized as a promising target antigen for improving the breadth and durability of influenza vaccines, yet NA-based mRNA vaccines remain comparatively unexplored relative to hemagglutinin-based approaches. Here, we applied a computationally guided NA antigen-design strategy to seasonal H3N2 influenza viruses and investigated whether epitope-guided optimization could improve the immunogenicity and protective efficacy of NA-based mRNA vaccines. NA-D3, an NA antigen designed based on the evolutionary characteristics of circulating H3N2 viruses, was first evaluated in an mRNA vaccine. This vaccine provided partial protection against H3N2 challenge and reduced viral replication in the lungs, although detectable neuraminidase inhibition (NAI) activity was not observed. To further improve NA antigen immunogenicity, predicted B- and T-cell epitopes were incorporated into the antigen design process, resulting in three sequentially optimized NA candidates, F0, F1, and F2. In a subsequent challenge experiment, F1 and F2 conferred complete protection, achieving 100% survival and significantly lower viral RNA levels and infectious viral titers in the lungs compared to controls. The optimized NA mRNA vaccines also induced detectable NAI activity against heterologous H3N2 viruses, albeit at relatively modest levels. Collectively, these findings demonstrate the protective potential of NA-based mRNA vaccination against H3N2 influenza viruses and establish iterative, epitope-guided strategy in NA antigen optimization – integrating computational prediction with experimental validation – as an effective approach for developing broadly protective NA-based mRNA vaccines.

## Introduction

Seasonal influenza viruses continue to pose a substantial global health burden, and the continuous evolution and reassortment of influenza A viruses generate extensive genetic and antigenic diversity. These characteristics complicate the development of vaccines capable of providing broad and durable protection against antigenically diverse viral strains. Although hemagglutinin (HA) remains the primary target of conventional influenza vaccines, increasing evidence indicates that neuraminidase (NA), the second major surface glycoprotein of influenza A viruses, also represents an important target of protective immunity.

Influenza A viruses possess an eight-segmented negative-sense RNA genome encoding polymerase basic 1 and 2 (PB1 and PB2), polymerase acidic (PA), hemagglutinin (HA), nucleoprotein (NP), neuraminidase (NA), matrix protein (M), and non-structural protein (NS) (Bouvier and Palese, 2008). HA and NA play complementary roles in the viral life cycle, mediating viral entry and release, respectively (Bouvier and Palese, 2008). While HA undergoes substantial antigenic variation that enables immune escape (Bliss et al., 2024; Wang et al., 2022), NA generally exhibits greater sequence conservation and is subject to distinct evolutionary constraints (Gravel et al., 2010; Leonard et al., 2024; Skarlupka et al., 2021). NA-specific antibodies can inhibit neuraminidase activity, restrict viral spread, and reduce disease severity, supporting NA as an attractive target for broadly protective influenza vaccines (Eichelberger et al., 2018; Leonard et al., 2024; Skarlupka et al., 2021).

Recent advances in computational biology have enabled the systematic characterization of viral sequence diversity and identification of conserved, evolutionarily relevant, and potentially immunogenic regions. By integrating phylogenetic, evolutionary, and structural analyses, vaccine antigens can be rationally designed to represent the genetic diversity of circulating viruses while incorporating residues that may contribute to antigenicity. In our previous study, we established a computationally guided strategy for NA antigen design and demonstrated that NA-based mRNA vaccine candidates induced protective immunity against influenza virus challenge. Building on this approach, we sought to determine whether the same antigen-design strategy could be extended to the antigenically diverse H3N2 subtype and whether additional antigen optimization would be required.

In the present study, we applied the previously established NA antigen-design strategy to H3N2 influenza viruses. Initial H3N2 NA vaccine candidates were designed based on the evolutionary and structural characteristics of circulating H3N2 viruses and evaluated for their immunogenicity and protective efficacy in vivo. Although the initial candidates provided partial protection against H3N2 virus challenge, detectable NA-specific inhibitory activity was not observed, suggesting a need for further optimization of the antigen design. To address this limitation, H3N2-specific B- and T-cell epitopes were incorporated into the antigen-design process, resulting in optimized NA vaccine candidates. Their immunogenicity and protective efficacy were evaluated to determine whether epitope-guided antigen optimization could enhance functional NA-specific antibody responses without compromising protection. Collectively, this study establishes an iterative antigen-design strategy that integrates computational prediction with experimental validation and highlights the potential of epitope-guided optimization for developing more immunogenic and broadly protective NA-based influenza vaccines.

## Materials and Methods

### H3N2 NA sequence dataset and phylogenetic analysis

Complete NA sequences of human H3N2 influenza viruses collected since 1999 were retrieved from the National Center for Biotechnology Information (NCBI) (https://www.ncbi.nlm.nih.gov) and the Global Initiative on Sharing Avian Influenza Data (GISAID) (http://www.gisaid.org) (Shu and McCauley, 2017). To minimize the influence of uneven temporal and geographic sampling, three independent datasets were generated by random subsampling, containing 717, 716, and 716 NA sequences, respectively. Fifteen representative H3N2 vaccine strains were included as reference sequences: A/Moscow/10/1999, A/Fujian/411/2002, A/California/7/2004, A/Wisconsin/67/2005, A/Brisbane/10/2007, A/Perth/16/2009, A/Victoria/361/2011, A/Texas/50/2012, A/Switzerland/9715293/2013, A/Hong Kong/4801/2014, A/Singapore/INFIMH-16-0019/2016, A/Kansas/14/2017, A/Hong Kong/2671/2019, A/Cambodia/e0826360/2020, and A/Darwin/09/2021. For the subsequent optimization of NA vaccine antigens, a separate dataset comprising 31,625 complete N2 sequences collected between 2023 and 2024 was retrieved from NCBI and GISAID. Three independent subsets were generated by random sampling, with 457 sequences included in each subset. Multiple sequence alignments were performed using MAFFT v7.419 (RIMD, Japan) (Katoh and Standley, 2013). Time-resolved phylogenetic reconstruction was performed using BEAST v1.10.4 (BEAST Developers) (Suchard et al., 2018). A GTR+I+G nucleotide substitution model with gamma-distributed rate variation was combined with an uncorrelated lognormal relaxed molecular clock. Bayesian inference was performed using Markov Chain Monte Carlo (MCMC) sampling for 200 million iterations, with states recorded every 200,000 steps. MCMC convergence was assessed using Tracer v1.7.1 (BEAST Developers). Maximum Clade Credibility (MCC) trees were summarized with TreeAnnotator v1.10.5 (BEAST Developers) and visualized with FigTree v1.4.4. A tanglegram analysis was performed to compare the evolutionary relationships between HA and NA and to identify discordant phylogenetic patterns suggestive of reassortment. Site-specific selective pressure was evaluated using the Datamonkey web server. The ratio of nonsynonymous to synonymous substitutions (d*N*/dS) was used to characterize selection pressure, with values below and above 1 interpreted as purifying and positive selection, respectively. Positively selected sites were identified using fast unconstrained Bayesian approximation (FUBAR) and mixed-effects model of evolution (MEME). For FUBAR, sites with a posterior probability ≥ 0.9 were considered significant, whereas MEME sites were selected using a significance threshold of *p* ≤ 0.1.

### Structural characterization of NA sequences

Three-dimensional structures of H3N2 NA were generated using SWISS-MODEL and ColabFold based on the corresponding NA sequence. Amino acid substitutions identified from phylogenetic analyses were mapped onto the modeled NA structure using PyMOL Molecular Graphics System v2.5.5. Structural mapping was used to examine the spatial distribution and surface exposure of lineage-associated substitutions and to identify sequence features potentially relevant to antigenic variation and subsequent vaccine antigen design (Kim et al., 2018; Mortazavi et al., 2023; Rigsby and Parker, 2016).

### Prediction of B- and T-cell epitopes

To assess the potential immunological relevance of the sequence variation identified during antigen optimization, B- and T-cell epitopes were predicted using the consensus N2 NA sequence generated from the 2023–2024 sequence dataset. T-cell epitopes were predicted using NetMHCpan v4.1 (DTU Health Tech, Denmark) (Reynisson et al., 2020) and NetMHCIIpan v4.3 (DTU Health Tech, Denmark) (Nilsson et al., 2023) against reference panels of HLA class I and class II alleles. Peptides of 8-11 amino acids and 15 amino acids were evaluated for class I and class II binding, respectively. B-cell epitopes were predicted using complementary sequence- and structure-based approaches. BepiPred-3.0 (DTU Health Tech, Denmark) (Clifford et al., 2022) was applied to the primary sequence, whereas SEMA 3.0, ScanNET, DiscoTope-3.0, and SEPPA 3.0 were applied to the ColabFold-derived three-dimensional NA structures (Hoie et al., 2024; Shashkova et al., 2022; Yu et al., 2025; Zhou et al., 2019). For each prediction method, residues exceeding the default threshold recommended by the corresponding model were designated as predicted epitope residues (NetMHCpan and NetMHCIIpan, %rank ≤ 2.0; BepiPred-3.0, score ≥ 0.1512; DiscoTope-3.0, score ≥ −3.7; SEPPA 3.0, score ≥ 0.064; ScanNET and SEMA, model-defined default cut-offs). Predictions from the individual models were then integrated to identify residues supported by multiple independent predictors, with the number of supporting models used as an indicator of prediction confidence. To evaluate changes in predicted antigenic regions associated with sequence variation, epitope predictions were compared across consecutive time periods. For each amino acid substitution identified relative to the consensus sequence of the preceding period, predictions were repeated using the substituted sequence and structure. Changes in epitope prediction were classified as retention, loss, or acquisition of predicted epitopes. Residues consistently predicted by multiple prediction methods and maintained across evolutionary periods were prioritized as candidate antigenic determinants for vaccine antigen design.

### Design and production of NA mRNA

For the initial H3N2 vaccine evaluation, HA mRNA constructs were derived from A/Darwin/09/2021 (H3N2), whereas the NA mRNA construct corresponded to the consensus-based NA-D3 antigen. NA-D3 was generated by deriving a consensus sequence from the H3N2 NA sequence dataset, followed by the introduction of additional substitutions. The sequences were codon-optimized to improve translational efficiency (Stachyra et al., 2016) and synthesized by GenScript (Piscataway, NJ) before cloning into DNA template plasmids. For the subsequent antigen optimization study, NA-only mRNA constructs were generated by introducing sequence modification selected from the evolutionary, structural, and epitope analyses. The plasmid templates were linearized before in vitro transcription, during which N1-methylpseudouridine was incorporated in place of uridine. The resulting transcripts contained a 100-nucleotide poly (A) tail and Cap1 structures (Henderson et al., 2021, Kauffman et al., 2016). Following purification, the mRNAs were formulated in lipid nanoparticles (LNPs) at 1 mg/ml and stored at -80℃ until administration. The final LNP formulations showed an encapsulation efficiency above 85%, a polydispersity index below 0.2, pH 7.4 ± 0.5, endotoxin levels below 4 EU/ml and a zeta potential within ± 15 mV. Immediately before immunization, LNP formulations were diluted in chilled phosphate-buffered saline (PBS).

### Cells, viruses, and assessment of mRNA expression

HEK293T cells were cultured in Dulbecco’s modified Eagle’s medium (DMEM; Gibco), whereas MDCK cells were maintained in minimum essential medium (MEM). Both media were supplemented with 10% fetal bovine serum (FBS) and 1% penicillin-streptomycin. Cells were incubated at 37℃ in a humidified atmosphere containing 5% CO_2_. For evaluation of mRNA-mediated protein expression, HEK293T cells were seeded at 3 × 10^4^ cells per well and transfected with 200 ng of mRNA using Lipofectamine 3000 (Invitrogen) in Opti-MEM (Gibco) (Liu et al., 2025; Zhou et al., 2024). At 24 h post-transfection, cells were fixed with 4% paraformaldehyde and permeabilized. HA and NA protein expression was detected using NIBSC reference antisera (21/324 and 19/320, respectively), followed by incubation with Alexa Fluor 488-conjugated rabbit anti-sheep IgG H+L secondary antibodies (Jackson ImmunoResearch) for 1 h at room temperature (RT) in the dark. Nuclear DNA was counterstained with DAPI, and fluorescence signals were acquired using the Operetta Imaging System. For challenge experiments, mouse-adapted A/Switzerland/9715293/2013 (H3N2) was used under ABSL-2 conditions. For assessment of NA-specific antibody responses, A/Hong Kong/2671/2019 (H3N2), A/Kansas/14/2017 (H3N2), and A/Switzerland/9715293/2013, were used.

### Immunization and challenge in mice

Female C57BL/6 mice, 3 weeks of age, were obtained from Koatech Co., Ltd. (Republic of Korea) and maintained under sterile conditions at the Korea University College of Medicine. Two vaccination experiments were performed to evaluate the initial and optimized NA antigens (Fig. S1). In the initial evaluation, mice received two intramuscular immunizations at a 2-week interval. Mice in the monovalent HA or NA groups were administered 10 µg of the respective mRNA, while mice in the bivalent group received 20 µg of total mRNA comprising 10 µg each of HA and NA mRNA. An inactivated influenza vaccine (IIV; 10 µg) and PBS (100 µl) were included as positive and negative controls, respectively. Challenge was performed 2 weeks after the second immunization. For the subsequent antigen-optimization experiment, mice received two intramuscular doses of the redesigned NA mRNA candidates at a 3-week interval. Challenge was performed 3 weeks after the second immunization. Serum samples were collected one day before challenge for serological analyses. For both experiment, mice were intranasally infected with 5 MLD_50_ of mouse-adapted A/Switzerland/9715293/2013 (H3N2) (Choi et al., 2014; Liu et al., 2021; Wilson et al., 2016). Animals were monitored daily for 14 days after infection, including assessment of body weight and survival. A reduction in body weight of ≥ 30% was defined as the humane endpoint (Brochut et al., 2024; Zhang et al., 2020).

### Quantification of viral replication in lungs

For assessment of viral replication, lung tissues were collected at 3 and 6 days post-infection (dpi) and homogenized in 1 ml of DMEM supplemented with 0.3% bovine serum albumin. Following clarification by centrifugation, the resulting supernatants were used for viral RNA and infectious virus measurement. Viral RNA was extracted from the lung homogenates and quantified by one-step qRT-PCR (Applied Biosystems) targeting the matrix (M) gene. Reverse transcription was performed at 48°C for 15 min, followed by an initial denaturation at 95°C for 10 min and 45 amplification cycles of 95°C for 15 s and 60°C for 1 min. Viral RNA copy numbers were determined using a standard curve. Infectious virus was quantified by plaque assays using MDCK cells. Ten-fold serial dilutions of samples were inoculated onto MDCK monolayers for 60 min at RT. Following removal of the inoculum, cells were overlaid with medium containing 2% agarose and incubated at 37°C with 5% CO_2_ for 72 h. Viral plaques were visualized by crystal violet staining and infectious titers were calculated.

### Measurement of neuraminidase inhibition (NAI) activity

NA-specific inhibitory antibody responses were measured using an enzyme-linked lectin assay (ELLA), according to a previously described method (Couzens et al., 2014; Kim et al., 2026). Briefly, fetuin-coated 96-well plates were used to measure NA activity. Sera were treated with receptor destroying enzyme (RDE) and heat-inactivated before analysis. Serially two-fold diluted serum samples were mixed with virus and transferred to the fetuin-coated plates. Following incubation, NA activity was detected using horseradish peroxidase-conjugated peanut agglutinin (PNA; Sigma Aldrich), followed by color development with O-phenylenediamine dihydrochloride (OPD; Sigma Aldrich). Absorbance was measured at 490 nm, and NAI activity was calculated as percentage reduction in NA activity relative to the virus control.

### Statistical analysis

Statistical analyses were performed using GraphPad Prism v10 (GraphPad Software, USA). Differences in viral RNA copy numbers and infectious viral titers between groups were evaluated using an unpaired two-tailed Student’s *t*-test. Statistical significance was defined as a *p*-value < 0.05.

## Results

### Phylogenetic analysis and lineage-associated mutations in H3N2 NA

Three independently subsampled datasets containing 717, 716, and 716 complete NA sequences from influenza A(H3N2) virus were analyzed using a Bayesian phylogenetic framework implemented in BEAST (v1.10.4). The maximum clade credibility (MCC) trees consistently resolved the H3N2 NA sequences into five distinct groups (Groups A–E), with comparable group distributions across the three datasets (Fig. 1). This consistency across independent subsets indicated that the observed phylogenetic structure was not substantially affected by random sampling.

Group A spanned the earliest and broadest temporal range. This group accounted for approximately 23% of sequences across the three datasets and exhibited the largest number of amino acid substitutions. These included A18S, L23F, V30I, C42F, N93D, G143V, H150R, V194I, E199K, G216V, K221E, P267T, V307I, Y310H, L370S, S372L, K385N, N387K, and Q432E, relative to the A/Moscow/10/1999. Group B accounted for approximately 22% of sequences and was characterized by eight amino acid substitutions: L81P, D93G, D147N, I215V, S367N, K369T, N402D, and I464L. Several of these substitutions co-occurred, with S367N/K369T and L81P/N402D, frequently observed as paired substitutions. Group C accounted for 20–24% of sequences and was characterized by five substitutions: E221D, S245N, S247T, T276K, and I380V. Group D accounted for 22–24% of sequences and was characterized by D339N and P468N. Notably, the recent vaccine strains A/Hong Kong/2671/2019, A/Cambodia/e0826360/2020, and A/Darwin/9/2021 clustered within Group D and exhibited nine additional substitutions: P126L, R150S, K220N, V303I, S315R, E344D, G346D, D463N, and N465S. Group E was the smallest group, accounting for 8–10% of sequences, and was mainly composed of viruses isolated from 2017 to 2019. Three characteristic substitutions, I176M, N329S, and P386S, were identified in this group.

The reference dataset included 15 historical and vaccine-related H3N2 strains: A/Moscow/10/1999, A/Fujian/411/2002, A/California/7/2004, A/Wisconsin/67/2005, A/Brisbane/10/2007, A/Perth/16/2009, A/Victoria/361/2011, A/Texas/50/2012, A/Switzerland/9715293/2013, A/Hong Kong/4801/2014, A/Singapore/INFIMH-16-0019/2016, A/Kansas/14/2017, A/Hong Kong/2671/2019, A/Cambodia/e0826360/2020, and A/Darwin/09/2021. These reference strains were distributed across the phylogenetic groups, reflecting the progressive evolution of H3N2 NA over the time. Sequence logo analysis further illustrated the predominant amino acid residues associated with each group and their temporal distribution (Fig. 1). Collectively, these findings demonstrated distinct lineage-associated patterns of amino acid variation across H3N2 NA sequences.

### HA–NA lineage associations and reassortment patterns

To assess the evolutionary association between HA and NA, phylogenetic trees were compared using tanglegram analyses across the three independently generated datasets (Fig. S2). The analyses revealed frequent discordance between the HA and NA lineages, indicating frequent reassortment between the two surface glycoprotein segments. The frequency of discordant HA–NA pairings varied substantially among NA lineages and across the three subsets (Table 1). Groups A showed the lowest frequency of reassortment, with only 2.5–3.0% of NA sequences showing discordant HA-NA pairings. In contrast, Groups B and C showed intermediate reassortment frequencies of 21.4–26.5% and 17.4–26.5%, respectively. Group D displayed a higher frequency of discordant pairings, ranging from 21.0–50.0% across the three subsets. The highest frequencies were consistently observed in Group E, with 73.6–98.6% of sequences showing discordant HA-NA lineage assignments.

The tanglegram illustrated that reassortment was not restricted to a single lineage pair but occurred among multiple combinations of HA and NA groups (Fig. S2). In particular, the extensive discordance observed in Groups D and E indicated that recent H3N2 NA lineages were frequently associated with HA genes belonging to different phylogenetic groups. Collectively, these analyses demonstrated substantial discordance between HA and NA evolutionary relationships, with reassortment frequencies differing according to NA lineage and sampling subset.

### Structural and evolutionary analysis of H3N2 NA

Structural mapping of recurrent amino acid substitutions revealed that most variable residues were located within the head domain of H3N2 NA (Fig. 2). Several recurrent substitutions were associated with changes in N-linked glycosylation (NLG) patterns, including the loss of NLG sites at residues 93, 329, and 402 and the acquisition of an NLG site at residue 245. The S367N/K369T substitutions were associated with the acquisition of a potential NLG at residue 367, while variation at residue 468 has been linked to antigenic changes (Moasser et al., 2018; Wan et al., 2019). Notably, recent vaccine strains exhibited a characteristic gain of NLG at residue 463, associated with the D463N/N465S substitutions (Gao et al., 2022). Despite these site-specific changes, the overall proportion of NLG sites remained relatively stable across the analyzed period. Such changes in NLG patterns may alter the accessibility of antigenic epitopes, potentially contributing to antigenic variation and immune escape (Powell and Pekosz, 2020; Wan et al., 2019).

To further identify the residues under evolutionary pressure, site-specific selection analyses were performed using MEME (*p* ≤ 0.1) and FUBAR (posterior probability ≥ 0.9). A total of 31 positively selected residues were identified (Table 2), of which 18 overlapped with recurrent mutation sites: 81, 93, 143, 147, 150, 199, 215, 221, 267, 303, 315, 329, 346, 380, 385, 386, 432, and 468. Among these overlapping residues, further consideration was given to those showing evidence of positive selection in at least two independent analyses or evolutionary periods. Accordingly, residues 147, 150, 303, 380, 385, 386, and 432 were excluded because repeated evidence of positive selection was not observed. Residues 143, 199, and 215 were also excluded, as the corresponding substitutions had become fixed following the emergence of Group A. Residue 81 was excluded because positive selection was detected only in different, non-overlapping evolutionary periods. Based on these criteria, residues 93, 267, 315, 329, 346, and 468 were ultimately selected for consensus sequence construction of H3N2 NA.

### Determination of consensus NA sequences

Consensus sequences for Groups A–E were constructed by integrating patterns of positive selection, recurrent mutations, amino acid frequencies, and phylogenetic relationships. In Group A, multiple substitutions were identified, but most were excluded owing to a lack of evidence for positive selection. Positive selection was identified at residues 267 within Group A and at residue 93 across the overall dataset. At residue 93, three amino acids—asparagine (N), aspartic acid (D), and glycine (G)—were observed, with D predominant within the group but G showing a higher frequency over the long-term period (G: 0.73% vs. D: 0.19%). Accordingly, the D-to-G substitution was incorporated into the A1 candidate. In contrast, the consensus residue at position 267 was retained, as no coexisting amino acid variants were observed. In Group B, none of the identified substitutions showed significant positive selection, and the B0 consensus sequence was therefore retained without additional modifications. In Group C, substitutions at residues 221, 245, 247, 267, and 380 showed no evidence of positive selection, whereas residues 329 and 468 showed significant positive selection within this group. At residue 329, threonine (T) was selected from the observed N, lysine (K), and T residues based on its potential to alter the NLG pattern. At residue 468, histidine (H) was selected from proline (P), leucine (L), and H based on its predominant frequency and previously reported association with antigenic drift, resulting in the C1 candidate (Wan et al., 2019). For Group D, three candidate sequences, D1–D3, were designed by incorporating substitutions identified in recent isolates and residues with potential functional or antigenic relevance. D1 included S315R, N329S, and G346D, which were observed in recent isolates. G346S was additionally incorporated into D2 based on the higher frequency of serine (S) at this position. D3 incorporated further substitutions, including E344K, D463N, and N465S, based on their potential associations with N-linked glycosylation and antigenic evolution (Galeone et al., 2024; Ge et al., 2022). D3 was selected for experimental evaluation because it incorporated the most recent substitutions while considering their potential functional and antigenic relevance. In Group E, substitutions at residues 176, 329, and 386 showed no evidence of positive selection, and therefore the E0 consensus sequence was retained without additional modifications (Table 3).

### Optimization of NA candidates based on epitope analysis

To further enhance the antigenic properties of the H3N2 NA vaccine antigen, sequence variations identified through phylogenetic analysis were integrated with predicted B- and T-cell epitopes. A/Thailand/08/2022 (H3N2), a vaccine strain used in this study, served as the template for the initial NA mRNA vaccine construct. Based on the consensus sequence analysis, K210R was introduced into the vaccine strain-derived sequence to generate the F0 candidate. The F1 candidate was generated by introducing the R400K and S331G into F0 based on recurrent sequence variation and predicted B-cell epitope information. F2 was further modified by incorporating a predicted T-cell epitope region spanning residues 307–312, including the K308R substitution. The predicted B- and T-cell epitopes were mapped onto the NA structure to assess their spatial distribution. These regions were primarily located on the exposed surface of the NA head domain. The vaccine strain, F0, F1, and F2 NA mRNAs were evaluated in mice to determine whether sequential antigen optimization improved immunogenicity and protective efficacy.

### Protective efficacy and NAI responses induced by NA-based mRNA vaccines

Protein expression of the mRNA constructs was confirmed by immunofluorescence assays in HEK293T cells (Fig. 3). Following H3N2 challenge, the bivalent mRNA group showed the highest protection (66.7%), followed by the IIV group (33.3%), whereas all mice in the mono-HA and mono-NA groups succumbed to infection (Fig. 4A and 4B). Consistent with the survival outcomes, mice receiving the bivalent mRNA vaccine exhibited less severe body weight loss and recovered their body weight during the observation period. Viral replication in the lungs was evaluated by qRT-PCR and plaque assays. At 3 dpi, the bivalent mRNA and IIV groups showed significantly lower viral RNA levels than the PBS group (*p* = 0.002 and *p* = 0.003, respectively) (Fig. 4C). Infectious viral titers measured by plaque assay were also significantly reduced in the bivalent mRNA group compared with the PBS group at 3 dpi (*p* < 0.001) (Fig. 4D). At 6 dpi, viral RNA levels remained relatively low in the bivalent mRNA group, although the differences were not statistically significant. Infectious viral titers showed variable patterns among the groups, without a consistent reduction associated with vaccination. Despite the observed protection, NAI activity was not detected in sera from the vaccinated groups.

The protective efficacy of the optimized NA mRNA candidates was evaluated in a second challenge study, with the vaccine strain, F0, F1, and F2 candidates designed as shown (Fig. 5A). The immunization interval was extended from 2 to 3 weeks. Mice were immunized with the vaccine, F0, F1, and F2 NA mRNA, with IIV and PBS included as positive and negative controls, respectively. Mice immunized with F1 and F2 showed complete protection, with 100% survival, followed by the IIV group with 75.0% survival, whereas all mice in the PBS group succumbed to infection. The vaccine and F0 groups showed 66.7% survival (Fig. 5B and 5C). Consistent with the survival outcomes, F1- and F2-immunized mice exhibited less severe body weight loss and recovered their body weight during the observation period. Viral replication in the lungs was further evaluated by qRT-PCR and plaque assays. At 3 dpi, viral RNA levels were significantly lower in all NA mRNA groups than in the PBS group (Fig. 5D). Infectious viral titers showed a similar trend, with significantly lower titers observed in the F0 and F2 groups than in the PBS group (*p* = 0.005 and *p* = 0.009, respectively) (Fig. 5E). At 6 dpi, viral RNA levels remained lower in the NA mRNA- and IIV-immunized groups than in the PBS group. Infectious viral titers were also lower in the vaccinated groups, although the differences were not statistically significant among the groups.

NA-specific immunogenicity was assessed using neuraminidase inhibition (NAI) assays. Against A/Hong Kong/2671/2019 (H3N2), NAI activity was generally limited, with inhibition below 50% across the immunized groups at a 1:10 serum dilution, although the F2 and IIV groups showed relatively higher inhibition than the other groups (Fig. 6A). Against A/Kansas/14/2017 (H3N2), detectable NAI activity was observed across the immunized groups, but inhibition generally remained below 50% and decreased with increasing serum dilution (Fig. 6B). Against the more distant A/Switzerland/9715293/2013 (H3N2) strain, NAI activity was further reduced, with most groups showing less than 50% inhibition even at the lowest serum dilution. The IIV group showed the highest activity, reaching approximately 60% inhibition at 1:10, whereas the NA mRNA-immunized groups showed lower levels of inhibition (Fig. 6C). Overall, the optimized NA mRNA vaccines induced detectable but relatively limited NAI activity against heterologous H3N2 viruses.

## Discussion

In our previous study, we developed a computationally guided approach for designing NA antigens and demonstrated that NA-based mRNA vaccine candidates generated using this strategy could provide protection against influenza virus infection (Kim et al., 2026). In current study, we extended this approach to H3N2 influenza viruses, which exhibit substantial antigenic and genetic diversity, to assess whether the previously established design strategy could be applied to this subtype and whether additional antigen optimization was necessary.

We first characterized the evolutionary features of H3N2 NA sequences circulating since 1999 and used these features to guide the design of the initial NA vaccine candidates (Fig. 1). Bayesian phylogenetic analysis identified five major NA lineages, characterized by distinct patterns of surface-exposed amino acid variation. Structural analysis showed that these variable residues were predominantly distributed across the NA head domain (Fig. 2). Despite the accumulation of sequence variation over time, the overall proportion of NLG sites in NA remained relatively stable throughout the analyzed period, as observed in seasonal H1N1 HAs (Kim et al., 2013). Six positively selected sites - 93, 267, 315, 329, 346, and 468 - were consistently identified by both MEME and FUBAR analyses, suggesting that these positions have been subject to sustained evolutionary pressure (Table 2). Together with recurrent substitutions that were structurally compatible with the NA protein, these features were incorporated into the design of the initial consensus-based NA antigen, D3. In parallel, analysis of HA-NA reassortment patterns revealed frequent heterologous segment combinations (Table 1), with particularly between Groups D and E (Fig. S2), suggesting relatively flexible HA-NA pairing dynamics among circulating H3N2 viruses.

Although the initial NA-D3 vaccine provided measurable protection following H3N2 challenge, NAI responses were not detected in vaccinated mice (Fig. 4). Thus, the protective effect observed with NA-D3 could not be readily attributed to a detectable serum NAI response. This finding suggested that the initial antigen design could be further refined to improve the induction of measurable antigen-specific antibody responses. Importantly, the absence of detectable NAI despite protection also suggested that the protective effect of NA vaccination could not be fully explained by serum NAI activity alone. To address this limitation, we incorporated H3N2-specific epitope information into the antigen-design process. Sequence variation identified through evolutionary analysis was considered together with predicted B- and T-cell epitopes to generate the optimized NA candidates, F0, F1, and F2 (Fig. 5A). Rather than introducing entire epitope sequences, specific amino acid modifications were selected based on predicted immunologically relevant regions and evolutionary characteristics. This stepwise design allowed the effects of progressive antigen modification to be evaluated experimentally. All mRNA constructs showed robust protein expression in vitro (Fig. 3), suggesting that differences observed in vivo were unlikely to be attributable to major differences in antigen expression.

The challenge experiments provided evidence that this optimized antigen-design strategy could improve protective efficacy. In the initial experiment, the bivalent mRNA vaccine showed the highest protection among the mRNA groups and performed comparably to IIV, with reduced body weight loss and lower levels of viral RNA and infectious virus in the lungs (Fig. 4). These findings indicate that NA-directed immunity can contribute to protection against H3N2 infection, even when serum NAI is not readily detectable. In the second experiment, the F1- and F2-immunized mice showed 100% survival, compared with 66.7% survival in the vaccine and F0 groups and 75.0% survival in the IIV group (Fig. 5C). The complete protection observed with F1 and F2 was accompanied by less severe body weight loss and subsequent recovery of body weight. Consistent with these findings, viral RNA levels were significantly reduced in the F0, F1, and F2 groups at 3 dpi, while infectious viral titers were significantly lower in the F0 and F2 groups than in the PBS group (Fig. 5D and 5E). These results indicate that the optimized NA candidates provided improved protection against H3N2 challenge relative to the unmodified vaccine strain.

The relationship between NAI activity and protection is also noteworthy. Unlike the initial NA-D3 vaccine, which did not induce detectable NAI activity, the F1 and F2 candidates achieved complete protection despite relatively limited NAI activity against heterologous H3N2 viruses (Fig. 6). Against A/Hong Kong/2671/2019 and A/Kansas/14/2017, NAI activity was detectable in several NA mRNA-immunized groups but generally remained below 50% and declined with increasing serum dilution. NAI activity was further reduced against the more distant A/Switzerland/9715293/2013 strain, with the IIV group showing the highest inhibition. The magnitude of inhibition did not fully correspond to the degree of protection. This discrepancy suggests that NA-mediated protection may involve immune mechanisms beyond neuraminidase inhibition. Fc-mediated antibody effector functions, including antibody-dependent cellular cytotoxicity, antibody-dependent cellular phagocytosis, and complement-mediated activity, may contribute to antiviral protection but were not directly evaluated in the present study (Boudreau and Alter, 2019; Padilla-Quirarte et al., 2019). Additional assessment of these functional antibody responses, together with NA-specific cellular immunity, will be necessary to clarify the mechanisms responsible for protection induced by NA-based mRNA vaccination.

An important feature of the present study is the iterative nature of the antigen-design strategy. The initial NA-D3 antigen was first evaluated based on evolutionary and structural information, and its protective efficacy and immunogenicity were assessed experimentally. The absence of detectable NAI despite measurable protection provided a rationale for further antigen refinement through incorporation of epitope information. The resulting F0, F1, and F2 candidates were then evaluated again for immunogenicity and protective efficacy, demonstrating that experimental findings can inform subsequent antigen-design decisions. Although a complete computational-experimental loop was not implemented in the present study, these results support an iterative framework in which evolutionary, structural, and epitope-based predictions are combined with experimental immunological data to progressively refine NA antigen design.

Several limitations should be considered when interpreting the improved immune responses observed with the optimized candidates. First, two experimental variables were changed between the initial and subsequent vaccination studies: the NA antigen sequences were optimized, and the immunization interval was extended. Therefore, the individual contribution of antigen optimization and the extended immunization interval to the improved protective efficacy cannot be independently determined. However, F0, F1, and F2 were evaluated under the same 3-week immunization schedule, and F1 and F2 showed higher survival than F0, supporting a potential contribution of the antigen modifications to protective efficacy. Furthermore, F2, which incorporated predicted T-cell epitopes in addition to the B-cell epitope modifications present in F1, consistently showed higher NAI responses than F1 across the tested H3N2 strains. This finding suggests that the incorporation of predicted T-cell epitope may contribute to enhanced functional antibody responses. Nevertheless, because T-cell responses were not directly evaluated, the immunological mechanism underlying this difference remains to be investigated further. The present findings support the potential benefit of the optimized antigen designs, but controlled studies using identical immunization schedules will be necessary to distinguish the effects of antigen modification from those of immunization interval. Second, the relatively limited NAI responses observed in the optimized groups indicate that computationally guided antigen modification does not necessarily translate directly into strong functional antibody responses. Computational analyses are useful for identifying evolutionarily relevant residues and immunogenic regions, but they cannot fully recapitulate the complexity of antigen-antibody interactions, conformational effects, or antigenic phenotype (Kringelum et al., 2012). In particular, sequence conservation or predicted epitope exposure does not necessarily translate to functional antigenic conservation (Koel et al., 2013). The strain-dependent differences in NAI observed in the present study highlight the importance of experimentally validating computationally selected modifications. Third, the mouse model does not fully recapitulate the immune history and complexity of human influenza infection. Pre-existing immunity and immune imprinting can influence epitope targeting, antibody hierarchy, and the functional characteristics of NA-directed antibody responses (Knight et al., 2020; Lv et al., 2025). These factors may affect both the magnitude and breadth of vaccine-induced immunity in humans and may not be adequately represented in naïve mouse models. Therefore, further evaluation in models that reflect pre-existing influenza immunity will be necessary to determine whether the immune responses observed with the optimized NA antigens can be translated to human vaccination.

Overall, our findings demonstrate that a computationally guided NA antigen-design strategy can be extended to H3N2 influenza viruses and further refined through the incorporation of epitope information. The initial NA-D3 antigen provided protection despite the absence of detectable NAI, while the epitope-mediated optimization generated candidates that induced measurable NAI responses and, in the case of F1 and F2, complete protection against H3N2 challenge. Although the mechanisms underlying this protection and the individual contributions of antigen optimization and immunization interval remain to be determined, the findings support an iterative computational-experimental framework for NA antigen development. Incorporation of experimentally derived antigenicity and immune-response data into computational antigen design may further improve the immunogenicity, functional breadth, and protective efficacy of NA-based mRNA vaccines.

## Figures and Tables

**Fig. 1.**
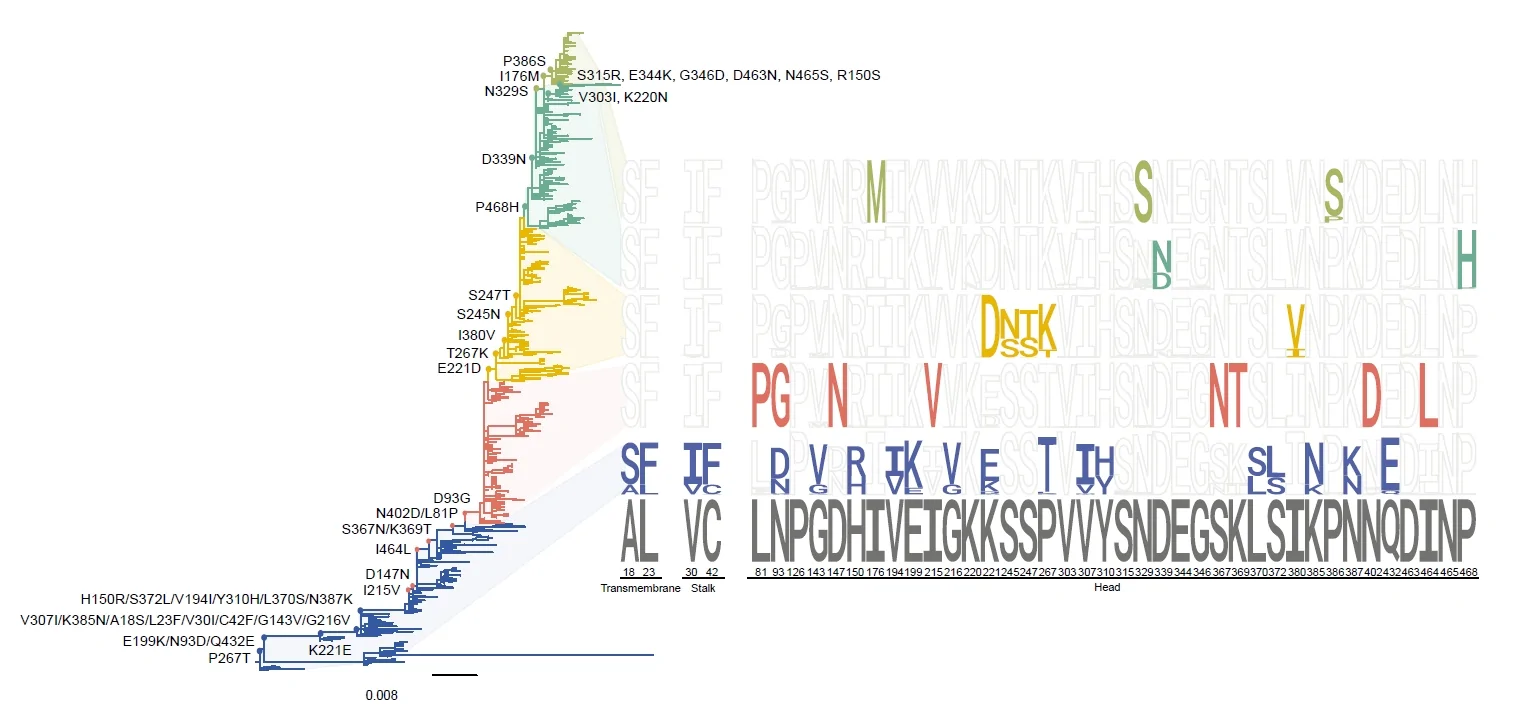
Phylogenetic and sequence variation analysis of NA genes from human H3N2 influenza viruses. The MCC tree of H3N2 NA genes were inferred using a Bayesian evolutionary framework. Phylogenetic branches are color-coded according to lineage classification: blue, Group A; red, Group B; yellow, Group C; blue-green, Group D; and light green, Group E. The adjacent sequence logo illustrates amino acid variation across NA positions relative to the A/Moscow/10/1999 (H3N2) reference sequence. Amino acid residues are displayed according to their relative frequencies across the phylogenetic groups, highlighting lineage-associated sequence variation in the NA protein.

**Fig. 2.**
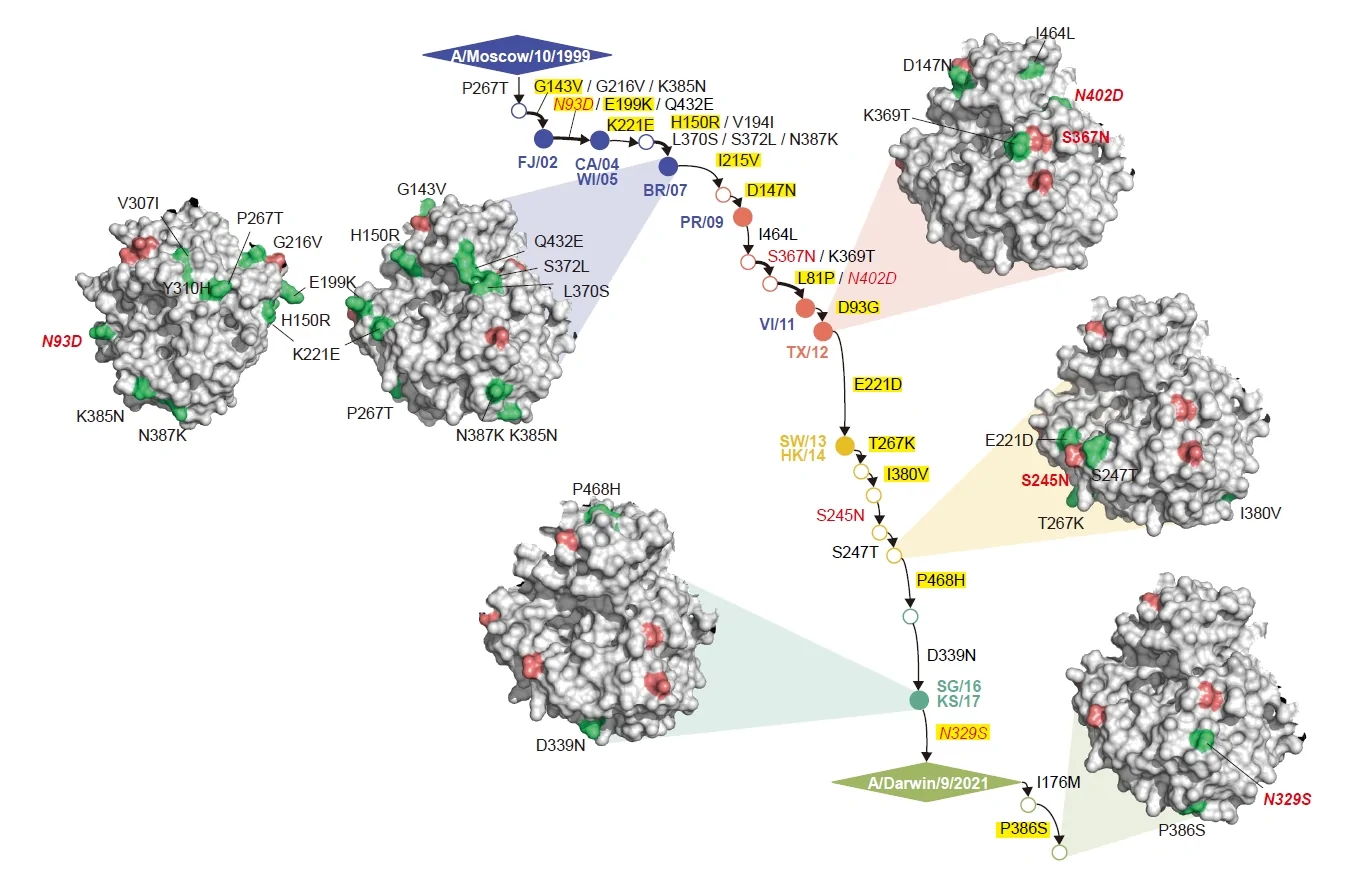
Structural analysis of representative H3N2 NA lineages. Representative NA sequences from phylogenetic lineages were mapped onto the evolutionary trajectory of H3N2 NA from the A/Moscow/10/1999 (H3N2) reference toward more recent circulating strains. Amino acid substitutions identified along the corresponding evolutionary branches are indicated, and representative NA structures are shown to visualize the spatial distribution of variable residues. Substitutions identified in the sequence analysis are highlighted in green, whereas N-linked glycosylation sites are indicated in red. The structural models illustrate the distribution of lineage-associated amino acid substitutions on the exposed surface of the NA head domain.

**Fig. 3.**
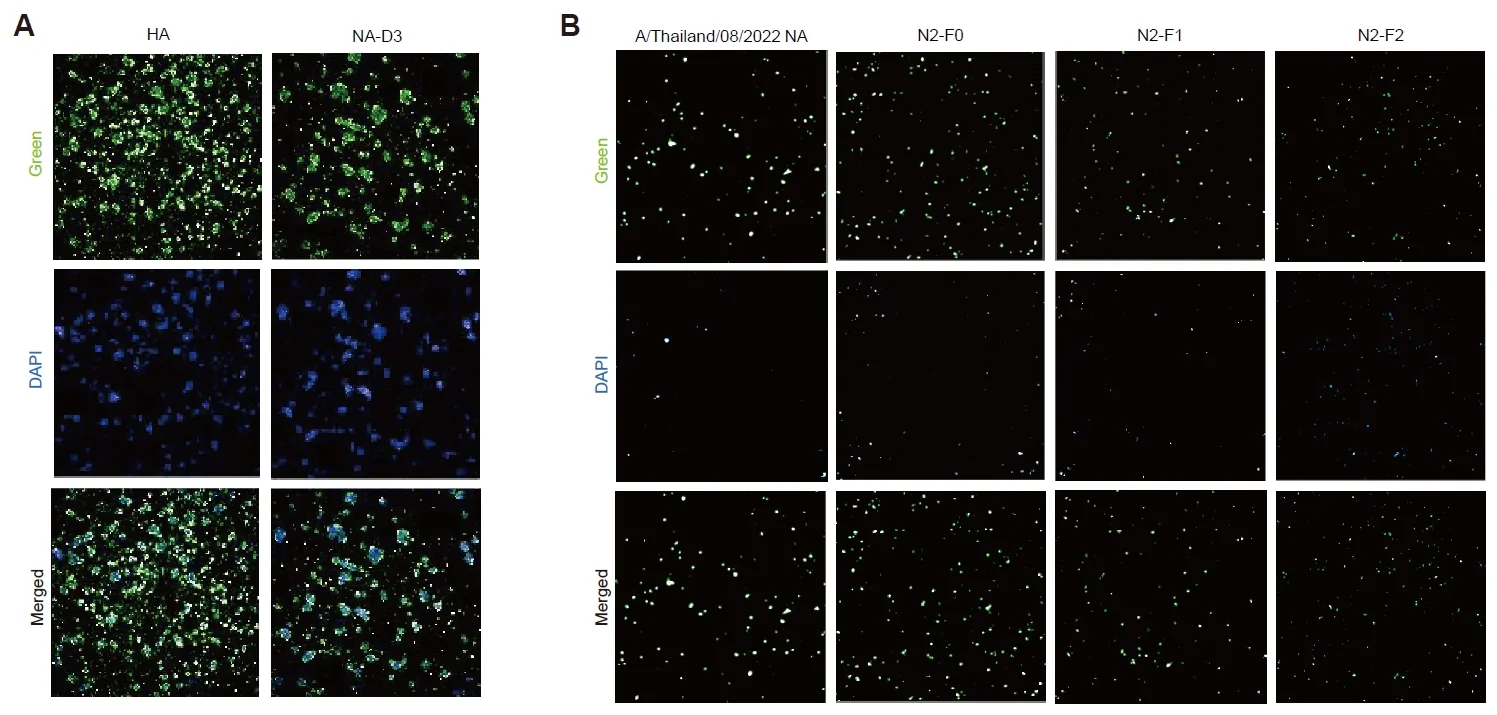
In vitro expression of initial and optimized mRNA constructs. Immunofluorescence assay (IFA) was performed to evaluate the protein expression in HEK293T cells transfected with the indicated mRNA constructs using Lipofectamine 3000. The cells were fixed 24 h post-transfection, and HA or NA proteins were detected using NIBSC antisera (21/324 and 19/320). Nuclei were counterstained with DAPI. Panels A and B show the expression of the initial and optimized mRNA constructs, respectively.

**Fig. 4.**
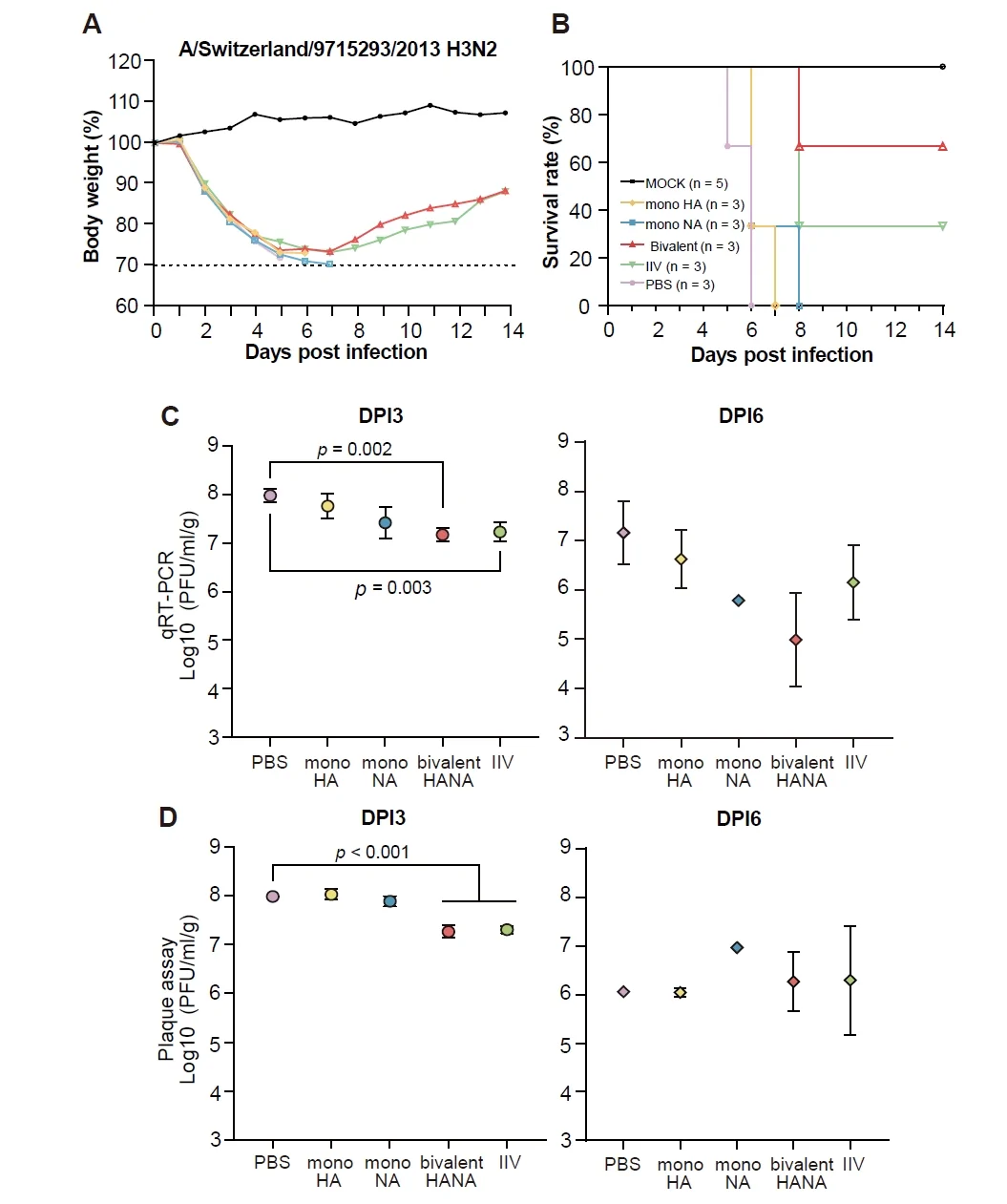
Protective efficacy of the initial mRNA vaccine candidates against H3N2 influenza virus challenge. (A) Body weight change and (B) survival of C57BL/6 mice following challenge with A/Switzerland/9715293/2013 (H3N2). Mice were monitored daily for 14 days post-infection (dpi). Three mice from each group were euthanized on 3 and 6 dpi for virological assays. (C) Viral RNA levels in lung tissues were quantified by qRT-PCR on 3 and 6 dpi. (D) Infectious viral titers in lungs were determined by plaque assay at the same time points. Statistical significance was assessed using an unpaired two-tailed Student’s *t*-test.

**Fig. 5.**
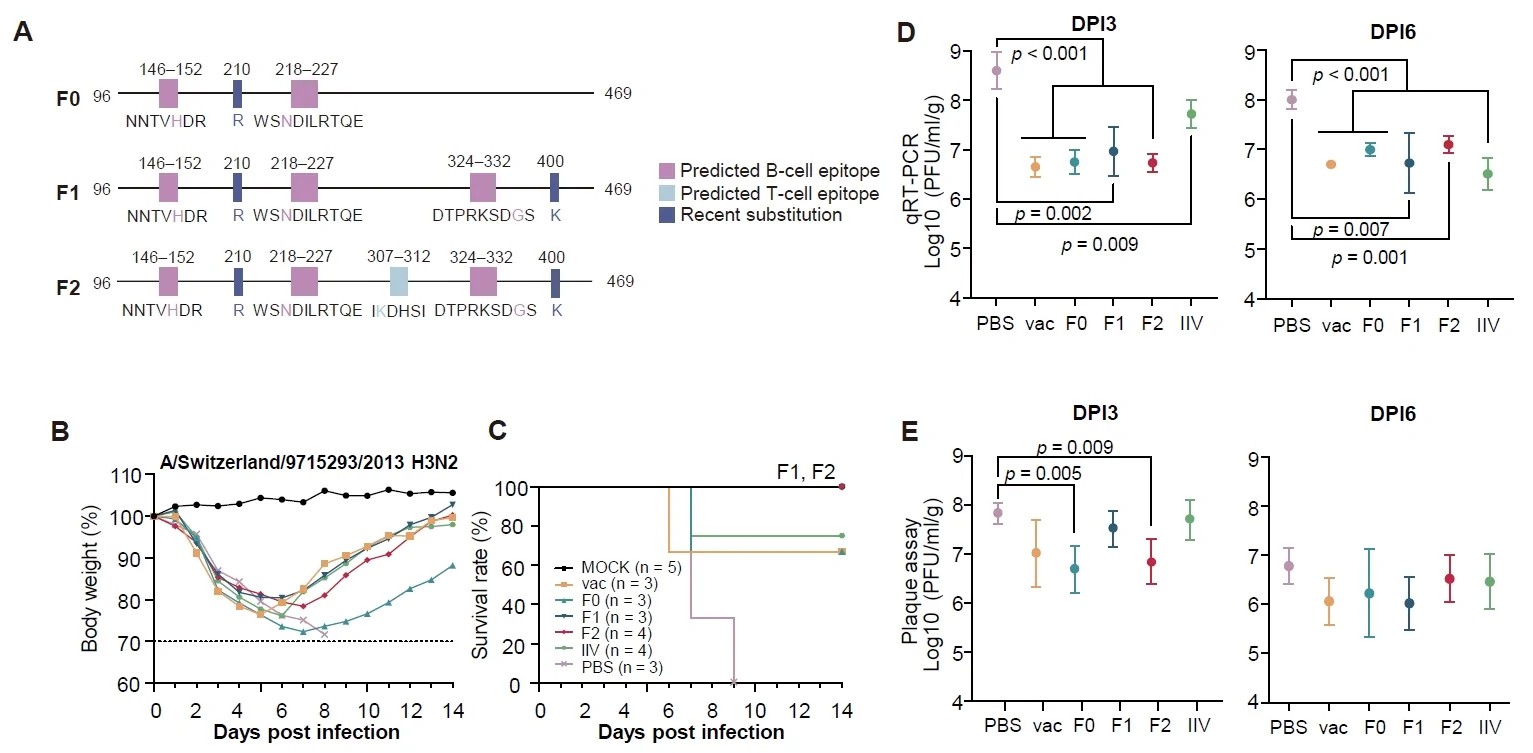
Design and protective efficacy of the optimized NA-based mRNA vaccine candidates against H3N2 influenza virus challenge. (A) Scheme of the sequential design of the F0, F1, and F2 NA mRNA antigens. Predicted B- and T-cell epitopes, recent substitutions are indicated according to the legend. F0 represents the initial redesigned NA antigen, while F1 and F2 were generated by sequential incorporation of additional sequence modifications and predicted epitope-associated residues. (B) Body weight change and (C) survival of C57BL/6 mice following challenge with A/Switzerland/9715293/2013 (H3N2). Mice were immunized with the vaccine-strain NA, F0, F1, or F2 NA mRNA, with IIV and PBS included as positive and negative controls, respectively. Mice were monitored daily for 14 dpi. Four mice from each group were euthanized on 3 and 6 dpi for virological assays. (D) Viral RNA levels in lung tissues were quantified by qRT-PCR on 3 and 6 dpi. (E) Infectious viral titers in lungs were determined by plaque assay at the same time points. Statistical significance was assessed using an unpaired two-tailed Student’s *t*-test.

**Fig. 6.**
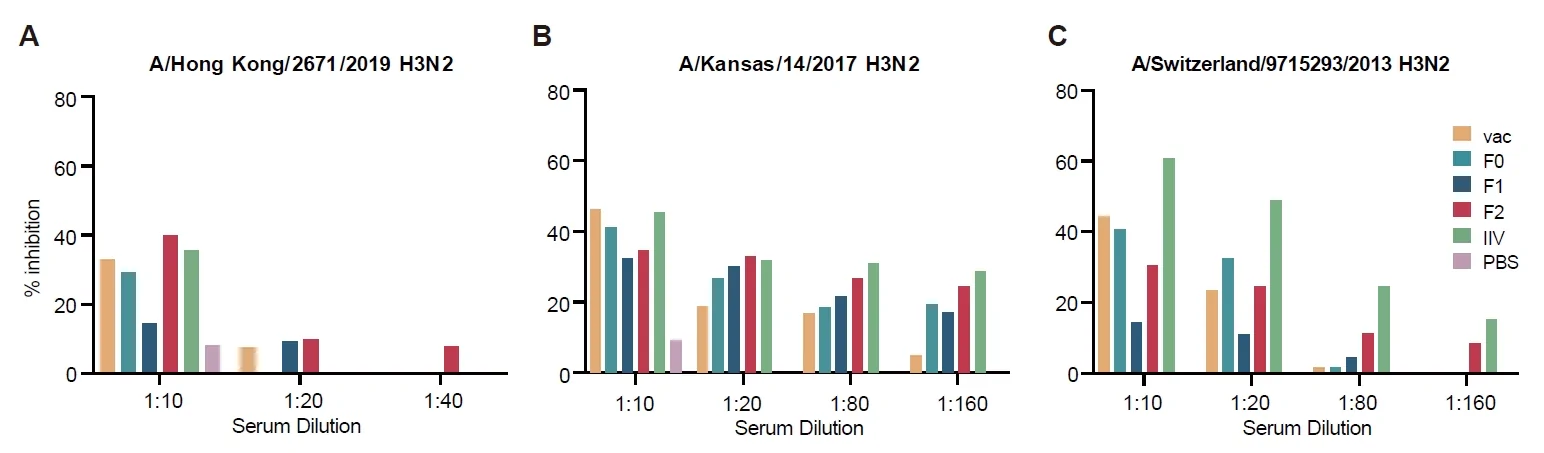
Neuraminidase inhibition activity induced by NA-based mRNA vaccination. Sera collected one day before viral challenge were serially diluted two-fold and evaluated for NAI activity using ELLA. NAI titers were assessed against (A) A/Hong Kong/2671/2019 (H3N2), (B) A/Kansas/14/2017 (H3N2), and (C) A/Switzerland/9715293/2013 (H3N2).

**Table 1.** Reassortment frequencies across H3N2 NA phylogenetic lineages

Subset	Lineage (NA)	No. of reassorted seqs	Total NA seqs	Frequency (%)
1	A	5	165	3.0
	B	34	159	21.4
	C	30	172	17.4
	D	34	162	21.0
	E	44	59	74.6
2	A	5	169	3.0
	B	35	159	22.0
	C	36	145	24.8
	D	59	171	34.5
	E	53	72	73.6
3	A	4	162	2.5
	B	43	162	26.5
	C	44	166	26.5
	D	77	154	50.0
	E	71	72	98.6

Three independent subsets were analyzed to assess the consistency of reassortment patterns across NA lineages. NA and HA lineages were assigned according to phylogenetic clustering, and isolates were classified as reassortment when their HA and NA genes belonged to different phylogenetic lineages. “No. of reassorted sequences” represents the number of such isolates identified within each NA lineage, while “Total NA sequences” represents the total number of NA sequences analyzed. Reassortment frequency was calculated as the proportion of reassorted sequences among the total NA sequences for each lineage.

**Table 2.** Positive selection across H3N2 NA phylogenetic lineages

Lineage	MEME	FUBAR
A	**81**, 147, **215, 267**,** 346**	221, **267**
B	**143**	**143, 329**
C	**143, 199, 329, 468**	**143, 329, 468**
D	**143**, **329**, 385	**81, 329, 346**, 380, **468**
E	-	**346**, 386
Entire dataset	150, **199, 215**, 303, **315, 329, 346, 385, 432, 468**	**93,** **215, 315, 329, 346, 468**

The table summarizes sites identified by MEME (*p* ≤ 0.1) and FUBAR (posterior probability ≥ 0.9) within each NA lineage and across the complete dataset. Sites highlighted in bold were detected in multiple independent analyses. Groups A–E correspond to the major phylogenetic NA lineages, while “Entire dataset” represents the combined analysis of all sequences. A dash (-) indicates the absence of detectable positive selection in the corresponding lineage.

**Table 3.** Summary of consensus sequences and candidate antigen designs

Group	Common mutations	Candidate	Incorporated substitutions	Basis for candidate design
A	N93D, G143V, H150R, V194I, E199K, G216V, K221E, P267T, V307I, Y310H, L370S, S372L, K385N, N387K, Q432E	A0	None	Group A consensus sequence
		A1	D93G	Positive selection and sequence variation
B	L81P, D93G, D147N, I215V, S367N, K369T, N402D, I464L	B0	None	Group B consensus sequence
C	E221D, S245N, S247T, T267K, I380V	C0	None	Group C consensus sequence
		C1	N329T, P468H	Positive selection, de-glycosylation and antigenic variation
D	D339N, P468H	D0	None	Group D consensus sequence
		D1	S315R, N329S, G346D	Substitutions observed in recent isolates
		D2	D1 + G346S	Higher frequency of S at residue 346
		D3	D2 + E344K, D463N, N465S	Recent variable and potential relevance to viral fitness, host adaptation, glycosylation, and antigenic variation
E	I176M, N329S, P386S	E0	None	Group E consensus sequence

A0–E0 represent the consensus sequences for each sequence group. Modified candidates were designed by incorporating selected substitutions identified through positive selection analyses, recent sequences variation, and their antigenic relevance. Group B and E retained their consensus sequences, whereas A1, C, and D1–D3 were generated with selected substitutions. Among the D group candidates, D3 was selected for experimental evaluation based on the incorporation of recent substitutions and their potential relevance to viral fitness, host adaptation, and glycosylation.

## Supplementary Information

Fig. S1. Experimental design of the animal studies. (A) Initial animal study evaluating mRNA vaccines. Mice received prime and boost immunizations on days 0 and 14, respectively, followed by serum collection on day 27 and viral challenge on day 28. Lung tissues were collected at 3 and 6 dpi, and the remaining mice were monitored until termination on day 42. (B) Subsequent animal study evaluating the optimized NA mRNA antigens. Mice received prime and boost immunizations on days 0 and 21, respectively, followed by serum collection on day 41 and viral challenge on day 42. Lung tissues were collected at 3 and 6 dpi, and the remaining mice were monitored until termination on day 56. Group sizes and vaccine doses are indicated in the Fig. IIV, inactivated influenza vaccine; PC, positive control; NC, negative control.

Fig. S2. Reassortment patterns between HA and NA genes in H3N2 viruses. Tanglegrams illustrate the phylogenetic relationships of HA (left) and NA (right) genes, with corresponding HA-NA pairs connected by lines. Viruses are assigned to five genetic clusters (A–E), and three independent datasets are shown (A, random 1; B, random 2; C, random 3). Connections between phylogenetic trees represent the HA and NA segments from the same isolate, with crossing connections indicating potential reassortment. Shared amino acid mutations are indicated at internal nodes, with sites under positive selection are highlighted in bold. The substitutions inferred to have occurred during the same period are enclosed in yellow box. The x-axes represent the number of mutations (left) and genetic divergence (right).
