# Supplementary material for: Epitope-guided optimization improves the protective efficacy of NA-based mRNA vaccines against seasonal influenza A(H3N2) virus
Source: J Microbiol. 2026 Oct 31;64(10):e2609004. doi: 10.71150/jm.2609004 (PMC13720382; doi:10.71150/jm.2609004)
Supplement: Supplementary file 1 [file jm-2609004-Supplementary-Fig-S1.pdf]

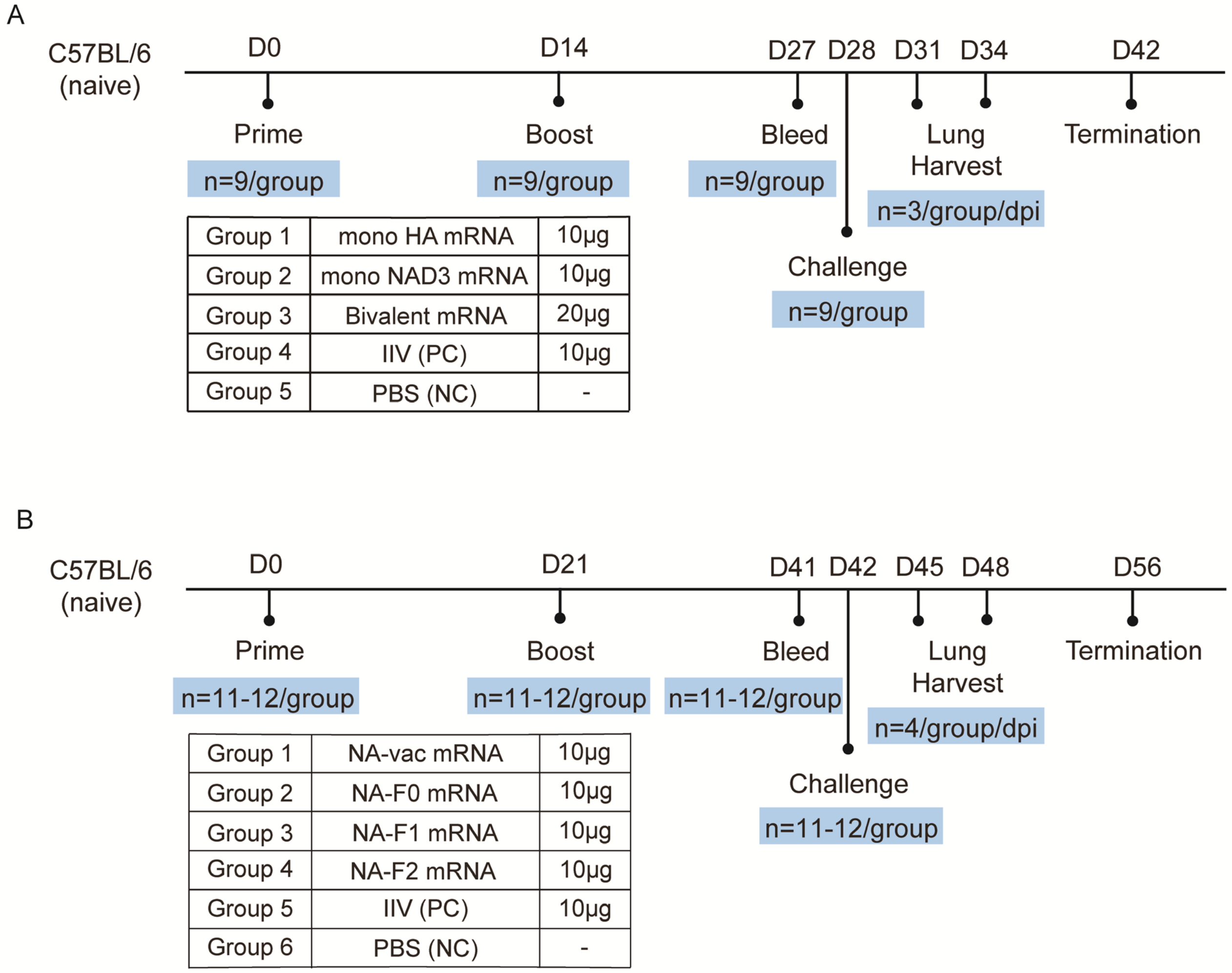


| | | |
|---|---|---|
| Group 1 | mono HA mRNA | 10μg |
| Group 2 | mono NAD3 mRNA | 10μg |
| Group 3 | Bivalent mRNA | 20μg |
| Group 4 | IIV (PC) | 10μg |
| Group 5 | PBS (NC) | - |

| | | |
|---|---|---|
| Group 1 | NA-vac mRNA | 10μg |
| Group 2 | NA-F0 mRNA | 10μg |
| Group 3 | NA-F1 mRNA | 10μg |
| Group 4 | NA-F2 mRNA | 10μg |
| Group 5 | IIV (PC) | 10μg |
| Group 6 | PBS (NC) | - |

**Fig. S1.** Experimental design of the animal studies. (A) Initial animal study evaluating mRNA vaccines. Mice received prime and boost immunizations on days 0 and 14, respectively, followed by serum collection on day 27 and viral challenge on day 28. Lung tissues were collected at 3 and 6 dpi, and the remaining mice were monitored until termination on day 42. (B) Subsequent animal study evaluating the optimized NA mRNA antigens. Mice received prime and boost immunizations on days 0 and 21, respectively, followed by serum collection on day 41 and viral challenge on day 42. Lung tissues were collected at 3 and 6 dpi, and the remaining mice were monitored until termination on day 56. Group sizes and vaccine doses are indicated in the Fig. IIV, inactivated influenza vaccine; PC, positive control; NC, negative control.
