# Supplementary material for: Epitope-guided optimization improves the protective efficacy of NA-based mRNA vaccines against seasonal influenza A(H3N2) virus
Source: J Microbiol. 2026 Oct 31;64(10):e2609004. doi: 10.71150/jm.2609004 (PMC13720382; doi:10.71150/jm.2609004)
Supplement: Supplementary file 2 [file jm-2609004-Supplementary-Fig-S2.pdf]

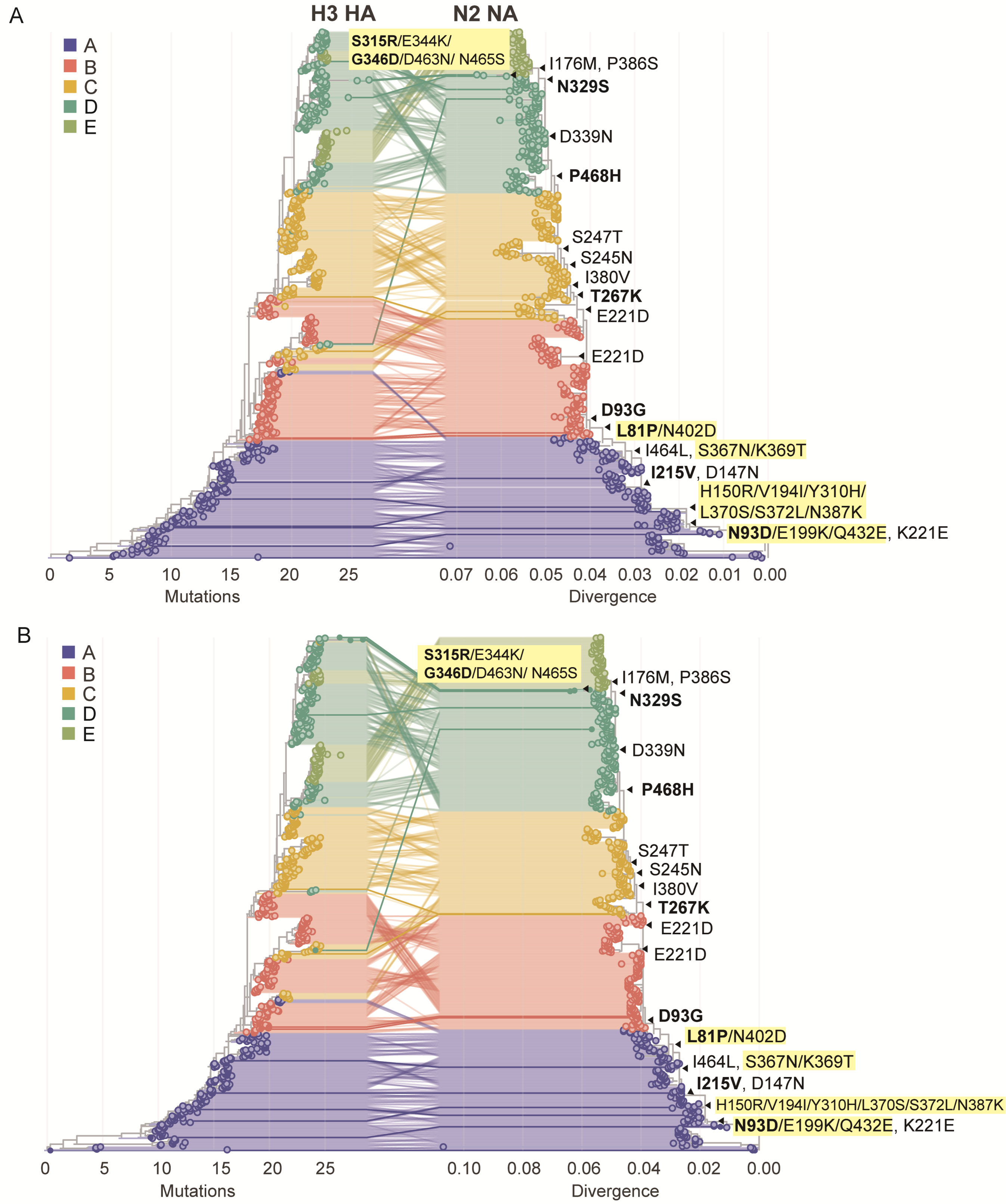

Fig. S2. Reassortment patterns between HA and NA genes in H3N2 viruses. Tanglegrams illustrate the phylogenetic relationships of HA (left) and NA (right) genes, with corresponding HA-NA pairs connected by lines. Viruses are assigned to five genetic clusters (A–E), and three independent datasets are shown (A, random 1; B, random 2; C, random 3). Connections between phylogenetic trees represent the HA and NA segments from the same isolate, with crossing connections indicating potential reassortment. Shared amino acid mutations are indicated at internal nodes, with sites under positive selection are highlighted in bold. The substitutions inferred to have occurred during the same period are enclosed in yellow box. The x-axes represent the number of mutations (left) and genetic divergence (right).

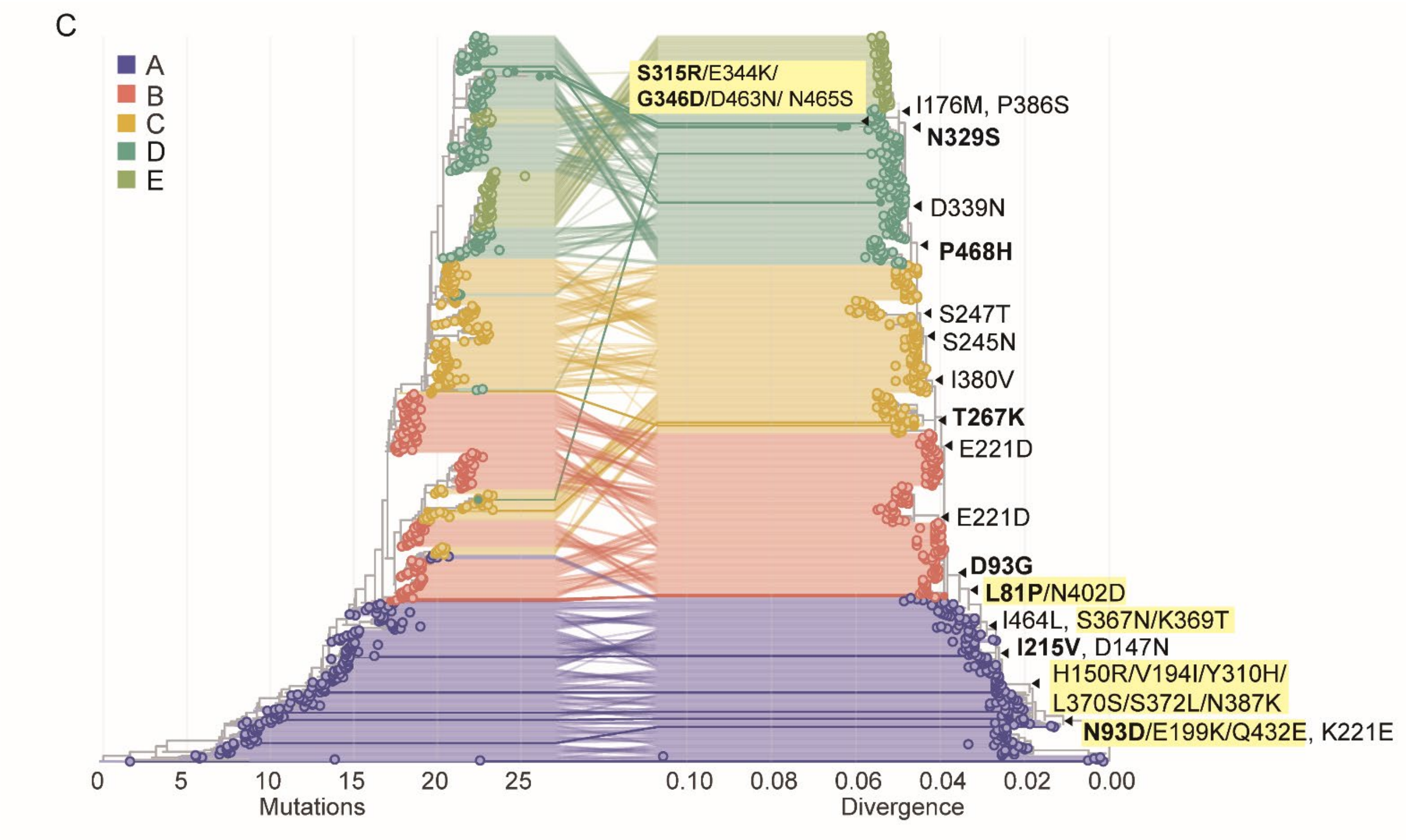

Fig. S2. (Continued)
